# Prenatal diagnosis of complete paternal uniparental isodisomy for chromosome 3: a case report

**DOI:** 10.1186/s13039-021-00569-8

**Published:** 2021-11-06

**Authors:** Xiufen Bu, Xu Li, Shihao Zhou, Liangcheng Shi, Xuanyu Jiang, Can Peng, Hongyu Li, Jun He

**Affiliations:** 1 Department of Genetics and Eugenics, Changsha Hospital for Maternal and Child Health Care, Changsha, 410007 Hunan China; 2 Department of Basic Medicine, Yiyang Medical College, Yiyang, 413000 Hunan China

**Keywords:** Uniparental isodisomy 3, Imprinted genes, Prenatal diagnosis, SNP array

## Abstract

**Background:**

Uniparental disomy (UPD) is defined as an inheritance of two chromosomes from only one of the parents with no representative copy from the other. Paternal-origin UPD of chromosome 3 is a very rare condition, with only five cases of paternal UPD(3) reported.

**Case presentation:**

Here, we report a prenatal case that is only the second confirmed paternal UPD(3) reported with no apparent disease phenotype. The fetus had a normal karyotype and normal ultrasound features throughout gestation. Copy neutral regions of homozygosity on chromosome 3 were identified by single nucleotide polymorphism (SNP) array. Subsequent SNP array data of parent–child trios showed that the fetus carried complete paternal uniparental isodisomy (isoUPD) of chromosome 3. The parents decided to continue with the pregnancy after genetic counseling, and the neonate had normal physical findings at birth and showed normal development after 1.5 years.

**Conclusions:**

These findings provided further evidence to confirm that there were no important imprinted genes on paternal chromosome 3 that caused serious diseases and a reference for the prenatal diagnosis and genetic counseling of UPD(3) in the future.

## Background

Uniparental disomy (UPD) is a rare condition defined as an inheritance of both chromosomes from only one parent without the presence of a representative copy from the other parent. The inheritance of a pair of homologous chromosomes from one parent is termed uniparental heterodisomy (hetUPD). If identical copies of one chromosome are inherited, the condition is termed uniparental isodisomy (isoUPD). Based on the original parent, UPD can be classified as maternal UPD (matUPD) or paternal UPD (patUPD); matUPD occurs more frequently than patUPD at a ratio of approximately 1:3. In matUPD, hetUPD is more common than isoUPD, while in patUPD, the frequencies of both conditions are almost equal [1]. UPD has been reported for all chromosomes except for the Y chromosome, but most frequently for chromosomes 1, 4, 16, 21, 22, and X. According to the latest scientific literature, the estimated incidence of UPD is 1/2000 live births [2]. The medical consequences of UPD may include autosomal recessive disease or abnormal imprinting. The phenotypes of autosomal recessive diseases can vary, with the majority of imprinting disorders presenting features that include aberrant pre- and/or postnatal growth, hypo- or hyperglycemia, abnormal feeding behavior in early and late childhood, behavioral difficulties, mental retardation, and precocious puberty [3]. Some reported cases involving children who had no clinical features and were found purely by chance through paternity testing.

UPD usually arises through meiotic non-disjunction with mitotic correction or gametic complementation. During the process, erroneous DNA replication, faulty DNA repair mechanisms and recombination can lead to genetic alterations. In fact, approximately 30% of UPD cases are associated with chromosomal aberrations that have frequencies of mosaic trisomy (39%), translocation (34%), and small supernumerary marker chromosomes (17%) [4]. Thus, it is difficult to predict the phenotype of UPD during prenatal diagnosis and it requires the accumulation of a large number of cases. According to the reported cases, the rate of UPD(3) was estimated to be 0.68% (31/4560), including 25 cases of complete UPD(3) [5]. Among the complete collection of known UPD(3) cases, there were 10 of unknown origin, 10 of maternal origin, and 5 of paternal origin. There is a clear underreporting of patUPD(3), which poses a significant challenge for prenatal genetic counseling. In this study, a prenatal patUPD(3) case with negative results of non-invasive prenatal screening (NIPT) and karyotype analysis was diagnosed by single nucleotide polymorphism (SNP) array and followed up.

## Case presentation

**Fig. 1:**
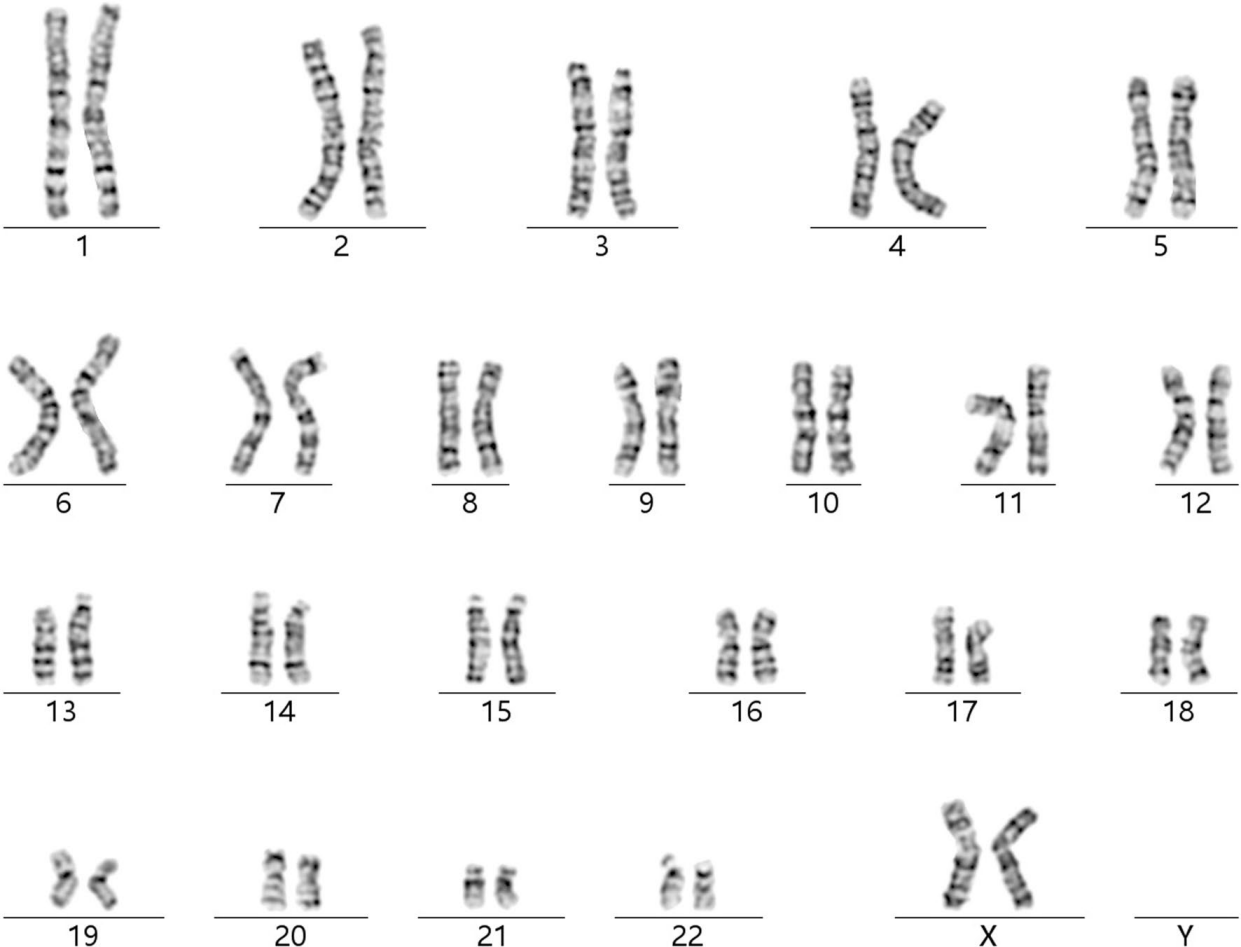
Normal karyotype of the fetus. The fetal amniotic fluid sample showed a normal 46, XX karyotype

**Fig. 2:**
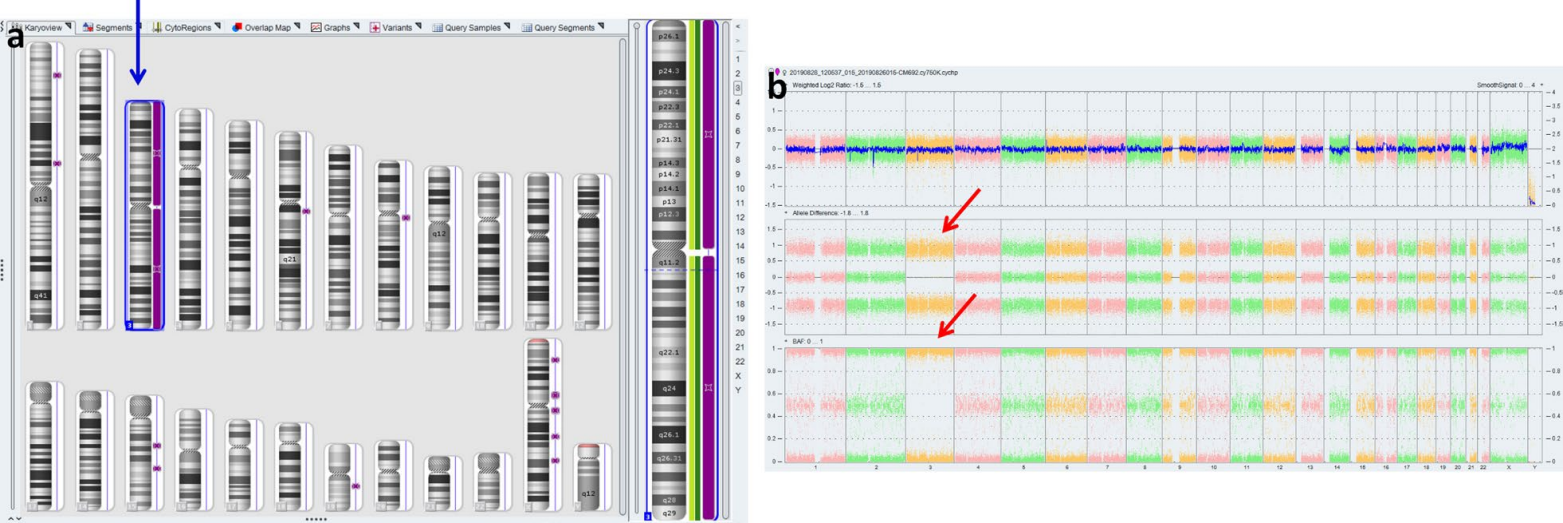
Regions of homozygosity (ROH) in chromosome 3 identified with SNP array analysis of the fetus. **a** ChAS revealed a complete ROH across the entire chromosome (purple rectangle, blue arrow). **b** A whole chromosome view clearly shows the copy neutral ROH on chromosome 3 in the fetus (red arrow)

**Fig. 3:**
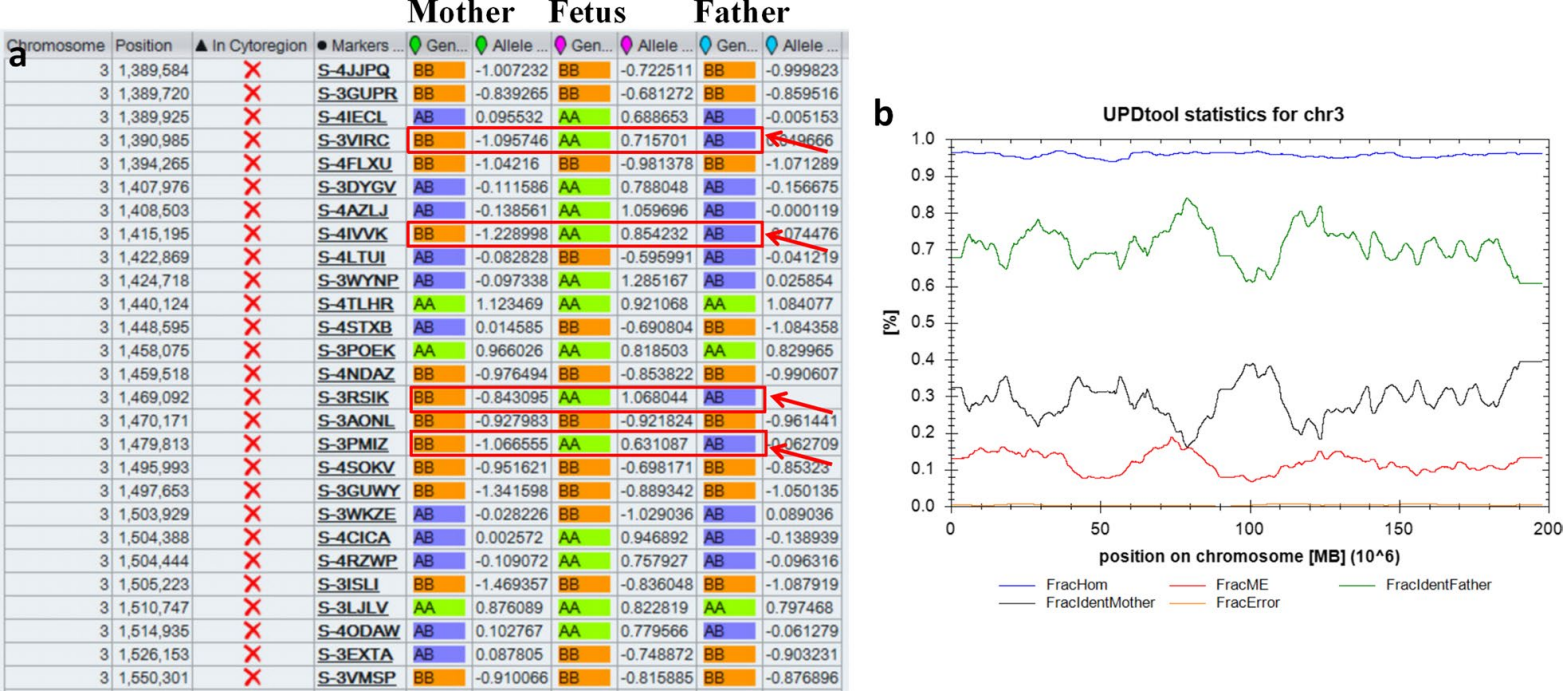
Complete paternal uniparental isodisomy (isoUPD) of chromosome 3. **a** ChAS software directly indicates that the UPD originated from her father after comparing the genotyping results between the fetus and her parents (red arrows). **b** Classification of UPD using the UPDtool showed the fetus was complete paternal isoUPD. FracHom (blue line) is the fraction of homozygous SNPs, FracME (red line) is the fraction of mendelian error SNPs, FracldentFather (green line) is the fraction of SNPs where the genotype is identical to the father, FracldentMother (black line) is the fraction of SNPs where the genotype is identical to the mother, and FracError (yellow line) is the fraction of errors. The UPDtool is available at: (http://www.unituebingen.de/uni/thk/de/f-genomik-software.html)

A healthy woman (gravida 4, induced abortion 2, missed abortion 1, para 0) was referred to the Department of Medical Genetics at Changsha hospital for maternal and child health care for opinion counseling due to advanced parental age (maternal age: 46; paternal age: 59), high-risk of Down's Screening in mid-pregnancy (T21 1:140), and adverse pregnancy history at 18 weeks and 3 days of gestation. Subsequent amniocentesis was arranged, and G-banding karyotype analysis (320 bands) of cultured amniocytes revealed a normal karyotype of 46, XX (Fig. 1). An Affymetrix CytoScan 750 K SNP array using uncultured amniocytes did not detect pathogenic copy number variants and revealed regions of homozygosity on chromosome 3 (Fig. 2a, b). The SNP microarray data of parent–child trios showed the fetus had complete paternal isoUPD(3), displayed by Chromosome Analysis Suite (ChAS) (Fig. 3a) and UPDtool statistics (Fig. 3b) respectively. The fetus presented no structural deformity during the whole pregnancy and the biparietal diameter, head circumference, femur length, humerus length, and abdominal circumference was consistent with gestational age when measured by ultrasound (Table 1). In addition, we mainly focused on placental mature grading to evaluate function of the placenta. Additionally, the parents had an unremarkable family history and refused further whole exome sequencing of uncultured amniocytes and decided to continue with the pregnancy after genetic counseling. At 36 weeks and 6 days of gestation, the pregnant woman had a Caesarean due to preeclampsia. A 2550 g female infant was delivered and had normal physical findings with an Apgar score = 10, 1 min after birth. Now, aged 1.5 years, the baby can walk independently and speak a few simple repeated words. Physical examination showed that she had achieved her appropriate developmental milestones and presented no physical abnormalities.

**Table 1:** The ultrasonic values that correspond to different gestational ages

Gestational age	BPD (mm)	HC (mm)	FL (mm)	HL (mm)	AC (mm)
24 W	59	222	44	37	197
28 W	70	262	54	47	240
32 W	79	297	63	56	280
36 W	93	338	67	58	320

*BPD* biparietal diameter, *HC* head circumference, *FL* femur length, *HL* humerus length, *AC* abdominal circumference, *W* week

## Discussion and conclusions

UPD usually arises from an error in the initial parental meiotic segregation, which results in the failure of two homologue chromosomes or sister chromatids to independently segregate into two daughter cells. As a result, the germ cell is disomic or nullisomic instead of being haploid. After fertilization, this zygote is trisomic or haploid. Trisomy rescue, monosomy rescue, and gametic complementation are the main reasons for UPD from an aneuploid to euploidy zygote [1, 6]. Trisomy rescue is the main mechanism, which was involved in at least 19% of the reported UPD cases and assumed to take place predominantly after the division of the zygote and loss of supernumerary chromosomes in trisomic cells is required to normalize the number of chromosomes in aneuploid cells [4]. About a third of the corrected cells exhibit UPD [7]. It is also possible that a monosomic cell can correct to disomy through a segregation error or duplication of the single chromosomal copy. IsoUPD is thought to form de novo in connection with a monosomy rescue process. PatUPD is the most common isoUPD, most likely duo to monosomy rescue [8]. UPD can also occur after fertilization of a nullisomic gamete by a disomic gamete (i.e., gametic complementation). The incidence of meiotic nondisjunction has been reported to increase with parental age [4]. In this case, the mother was 46 years old, and therefore, it was hypothesized that the complete isoUPD(3) was due to a maternal meiosis error that resulted in the fertilization of an egg without chromosome 3 by a normal sperm, followed by monosomic rescue that led to paternal isoUPD(3). It is noteworthy that the father was 57 years old, as the proportion of aneuploid sperm increases with age. The complete isoUPD(3) was due to a paternal meiosis error II could also have resulted in fertilization of a normal egg cell by a sperm with two identical chromosomes 3, followed by trisomy rescue to restore euploidy. In addition, meiotic errors can occur simultaneously during gamete formation and a sperm with two identical chromosome 3 could fuse with an egg missing chromosomes 3 and restore euploidy.

So far, only 25 cases of complete UPD(3) have been reported, including 10 cases of unknown origin, 10 cases of maternal origin, and 5 cases of paternal origin. Among the 10 cases of maternal UPD(3) with an abnormal phenotype, 6 had clear clinical phenotypes associated with chromosomal recessive disorders, 2 had abnormal chromosome karyotype, 1 had mosaic UPD, and detailed information was not available for 1 case. Among these six cases, the phenotypes included epidermolysis bullosa (*COL7A1*) [9, 10], Fanconi Bickel syndrome (*GLUT2*) [11], a congenital disorder of glycosylation type Id (*ALG3*) [12], GM1 gangliosidosis (*GLB1*) [13], and woolly hair/hypotrichosis (*LIPH*) [14]. Among the five reported cases of paternal UPD(3), three had a definite phenotype caused by single-gene disorders, one had no apparent disease phenotype, and one presented an abnormal karyotype. Among these three cases, the phenotypes involved Pierson syndrome (*LAMB2*) [15] and GM1 gangliosidosis (*GLB1* and *SLC25A38*) [16, 17]. Currently, only one case of paternal UPD of the entire chromosome 3 has been described with no apparent disease phenotype [18]. The male was identified serendipitously in the study through a whole genome linkage scan and did not display any obvious adverse phenotypic disorders at age 42. The male with a normal karyotype was 175 cm tall and showed no growth retardation; his father was 26 and the mother was 19 years old when he was born. We have now reported another case of UPD(3) without an obvious phenotype.

The present case was an isoUPD in which two identical copies of one homolog were inherited, which is at risk of homozygosity for chromosomal recessive disorders in the offspring of a heterozygous carrier. Therefore, when UPD is found during prenatal diagnosis, it is necessary to conduct further examination to exclude recessive gene pathogenic mutations. In this case, the fetus had normal ultrasound features throughout gestation and the parents refused the further examination. Although the child did not show any significant growth or developmental abnormalities until the age of 1.5 years, the risk of delayed manifestation of single gene disorders cannot be ruled out, and monitoring growth and development is required.

As a rare abnormality, UPD can lead to abnormal phenotypes associated with autosomal recessive disorders as well as through gene imprinting. At present, chromosomes 6, 7, 11, 14, 15, and 20 have been identified to cause imprinting diseases [19]. However, there are two paternally imprinted genes on chromosome 3 that have been predicted by bioinformatics: *ALDH1L* and *Z1C1*. If there were critical paternally imprinted genes on chromosome 3, they would not be functional due to non-expression of imprinted genes from the paternal disomic chromosomes, which would lead to imprinting diseases. This current case suggests that there is no important paternally imprinted gene on chromosome 3 that causes serious diseases. Nevertheless, we will continue to follow this case to confirm this hypothesis.

The case we reported was the second ascertained case of complete paternal isoUPD(3) with no genomic abnormality. Our study further showed that there were no important paternal imprinted genes that cause rare genetic disorders on chromosome 3 and provided a reference for future prenatal diagnosis and consultation for UPD(3).

## Data Availability

Not applicable to this article as no datasets were generated or analysed during the current study.

## Abbreviations

UPD: Uniparental disomy
hetUPD: Uniparental heterodisomy
isoUPD: Uniparental isodisomy
matUPD: Maternal uniparental disomy
patUPD: Paternal uniparental disomy
NIPT: Non-invasive prenatal screening
ROH: Regions of homozygosity
SNP array: Single nucleotide polymophism array
ChAS: Chromosome analysis suite
